# Supplementary material for: Highly Stretchable Graphene Scrolls Transistors for Self-Powered Tribotronic Non-Mechanosensation Application
Source: Nanomaterials (Basel). 2023 Jan 28;13(3):528. doi: 10.3390/nano13030528 (PMC9920215; doi:10.3390/nano13030528)
Supplement: Supplementary file 1 [file nanomaterials-13-00528-s001.zip › nanomaterials-1755578-supplementary.pdf]

Article

Highly Stretchable Graphene Scrolls Transistors for Self-Powered Tribotronic Non-Mechanosensation Application

Yanfang Meng [1,2,3]

[1] State Key Laboratory of Advanced Optical Communications System and Networks, School of Electronics Engineering and Computer Science, Peking University, Beijing 100871, China
[2] Beijing Institute of Nanoenergy and Nanosystems, Chinese Academy of Sciences, Beijing 100083, China
[3] School of Nanoscience and Technology, University of Chinese Academy of Sciences, Beijing 100049, China

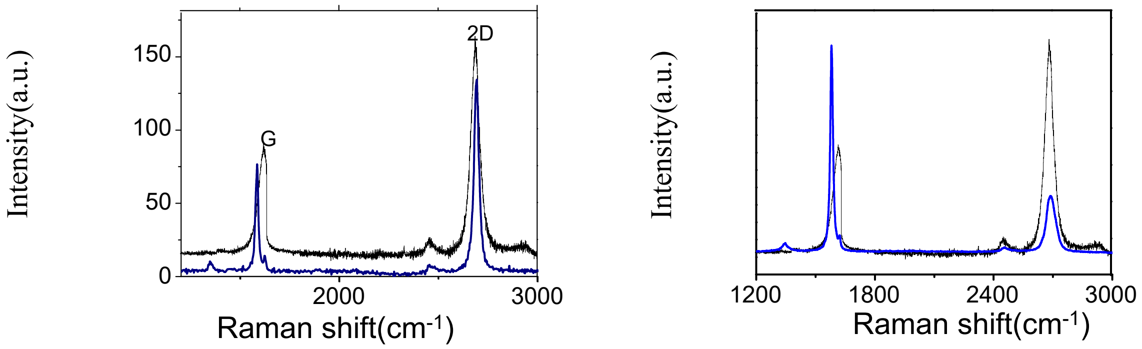

Figure S1. Left panel: Comparison of Raman spectrum of the CVD single-layer graphene (blank line) and monolayer graphene scroll (blue line). Right panel: Comparison of Raman spectrum of the monolayer graphene scroll (blank line) and trilayer graphene scrolls (blue line).

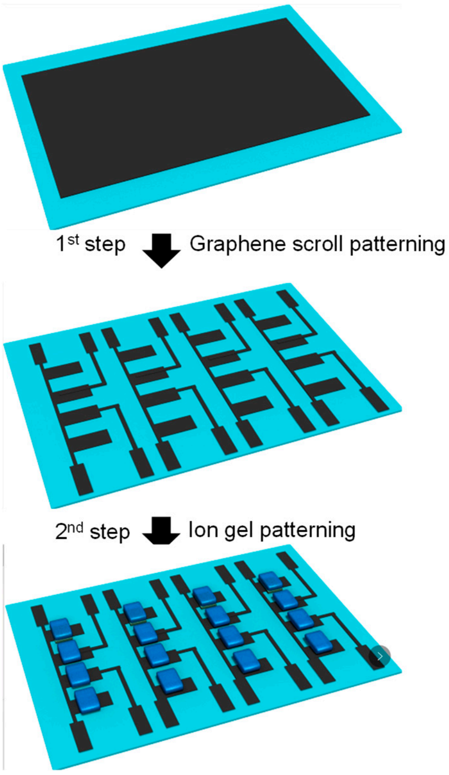

Figure S2. Fabrication procedure of the tribopotential-gated GSFET array.

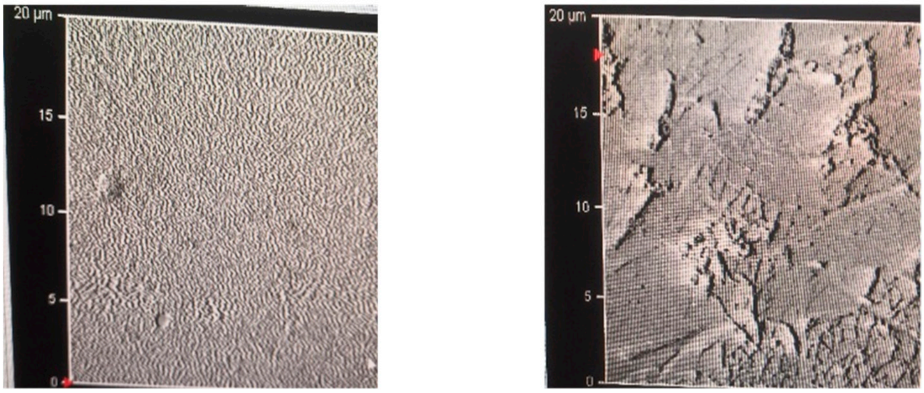

Figure S3. AFM (atom force microscopy) of the monolayer graphene (left panel) and trilayer graphene scrolls (right panel).

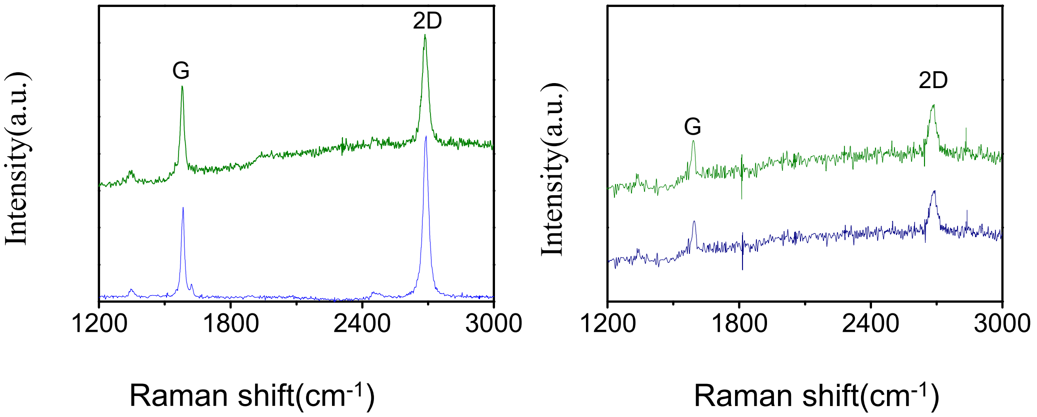

Figure S4. Raman characteristic of graphene under strain. Left panel: comparison of Raman spectrum of the CVD single-layer graphene under no strain (blue line) and under a 5% strain (green line). Right panel: comparison of Raman spectrum of the trilayer graphene scrolls under no strain (blue line) and under a 5% strain (green line).

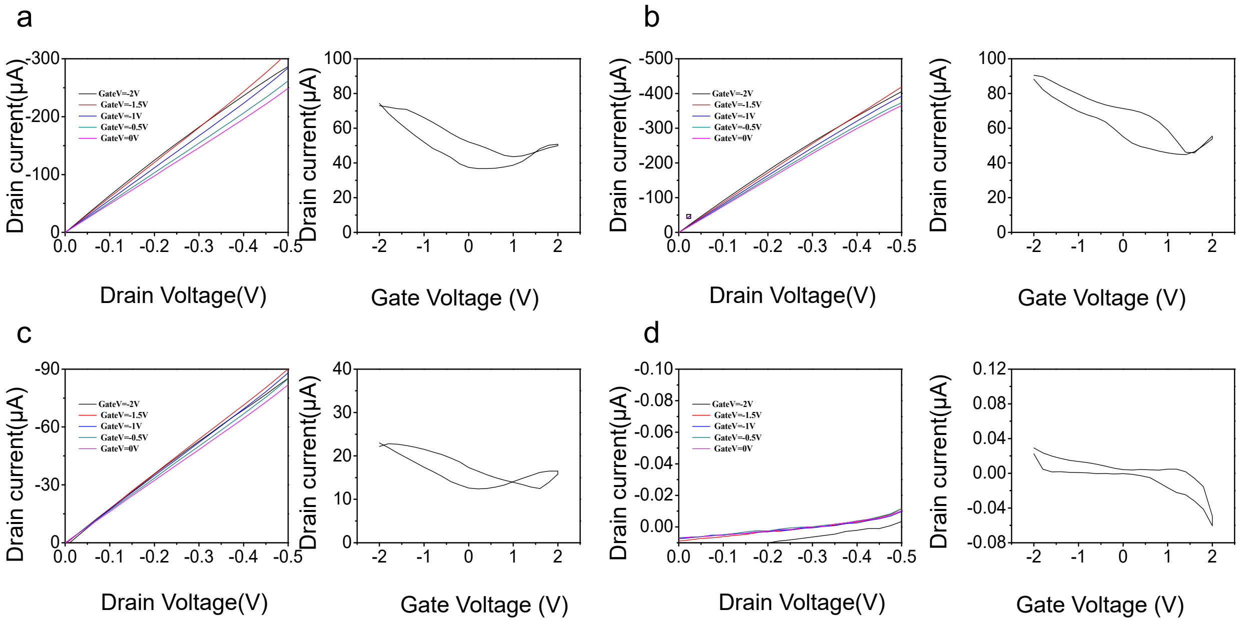

Figure S5. Electrical properties of GFET/GSFET under strain deformation (a) Output curve (left panel) and transfer curve (at the given V_D=0.1 V, right panel) of GSFET under no strain deformations. (b) Output curve (left panel) and transfer curve (at the given V_D=0.1 V, right panel) of GSFET under 5% strain deformations. (c) Output curve (left panel) and transfer curve (at the given V_D=0.1 V, right panel) of GFET under no strain deformations. (d) Output curve (left panel) and transfer curve (at the given V_D=0.1 V, right panel) of GFET under 5% strain deformations.

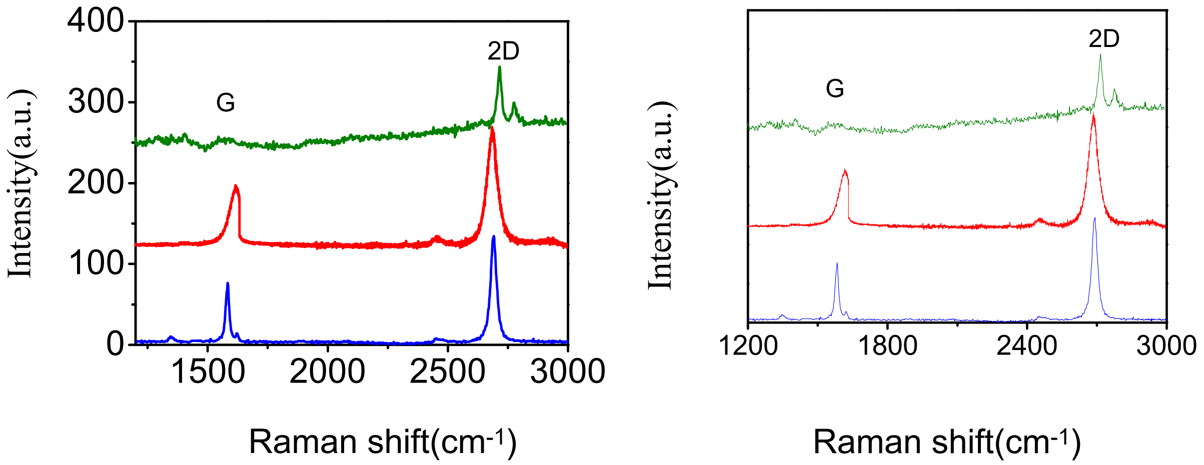

Figure S6. Left panel: comparison of Raman spectrum of the CVD single-layer graphene under room temperature (blue line), 30°C (red line) and under a 5% strain (green line). Right panel: comparison of Raman spectrum of the tri-layer graphene scrolls under room temperature (blue line), 30°C (red line) and under a 5% strain (green line).

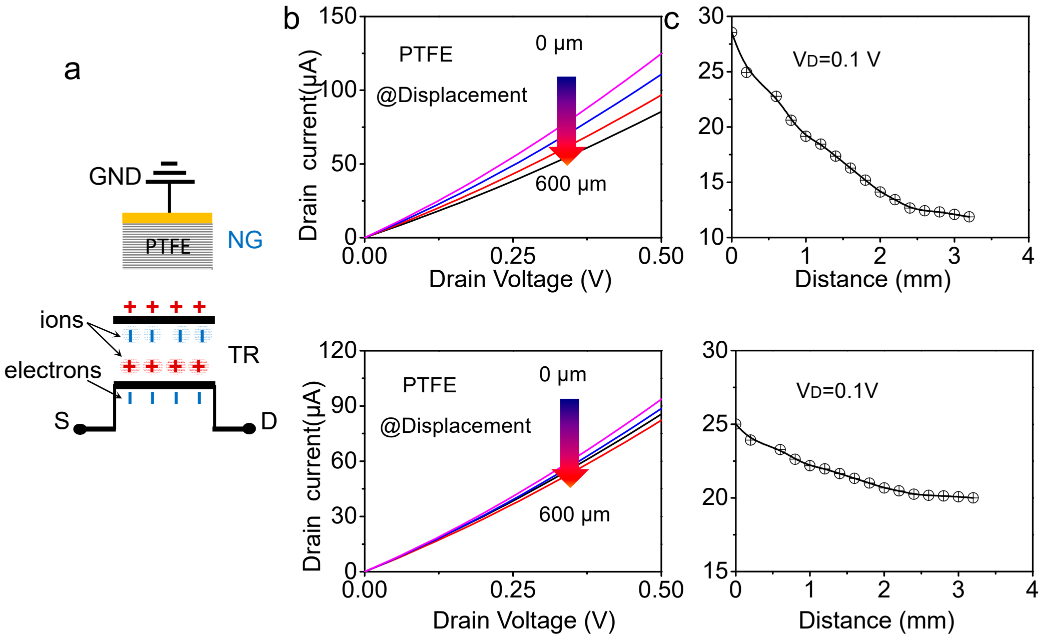

Figure S7. Triboelectrical performances of GFET/GSFET by tribo-potential between ion gel and PTFE (a) Circuit diagram of tribotronic GFET/GSFET sensor contact with PTFE. (b) Output curve (left panel) and transfer curve (at the given V_D=0.1 V, right panel) of GFET powered by tribopotential between ion gel and PTFE under no strain deformations. (c) Output curve (left panel) and transfer curve (at the given V_D=0.1 V, right panel) of GSFET powered by tribopotential between ion gel and PTFE under no strain deformations.

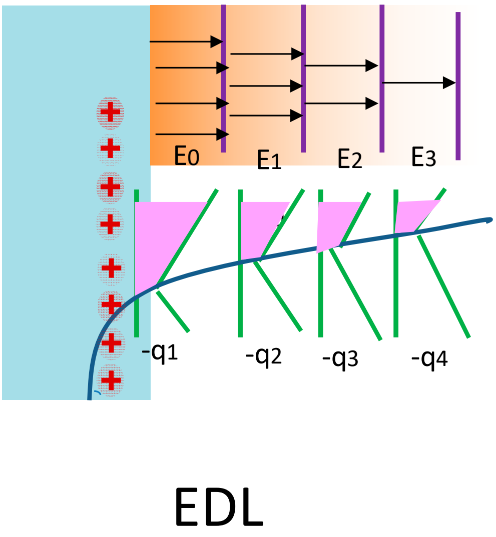

Figure S8. Schematic illustration of degradation of triboelectric responsiveness of trilayer GSFET comparing to monolayer graphene FET.

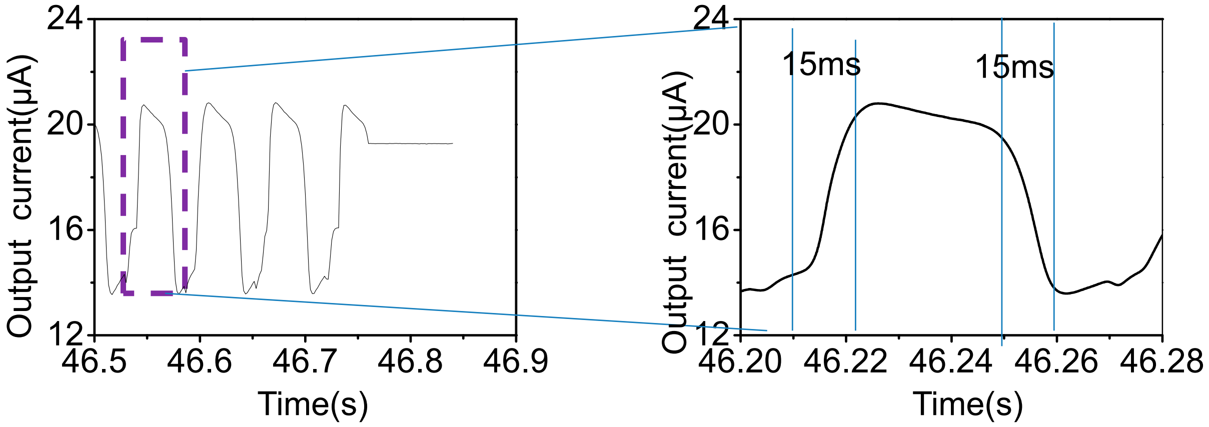

Figure S9. The I_D response time of the GSFET.
